# A putative lncRNA RBM26-AS1-encoded micropeptide promotes colon cancer progression

**DOI:** 10.1097/JS9.0000000000002834

**Published:** 2025-07-09

**Authors:** Qi Wu, Zhenling Ji

**Affiliations:** a School of Medicine, Southeast University, Nanjing, Jiangsu, China; b Department of General Surgery, Zhongda Hospital, Southeast University School of Medicine, Nanjing, Jiangsu, China; c Department of General Surgery, Nanjing Jiangbei Hospital, Nanjing, Jiangsu, China

**Keywords:** colon cancer, long non-coding rNAs (lncRNAs), micropeptide, tumour progression

## Abstract

**Background:**

The functional diversity and mechanistic complexity of long non-coding RNAs (lncRNAs) exert various regulatory roles in cancer, and they have traditionally been annotated as non-coding genes. Currently, the coding potential of lncRNAs is gradually being revealed; however, their validation and mechanisms of action in cancer remain largely unknown.

**Methods:**

The expression, prognosis, and function of *RBM26-AS1* in colon cancer were analyzed by bioinformatics, and its coding potential was predicted. The expression and localization of the RBM26-AS1 peptide were verified by constructing various fusion constructs, western blotting, and immunofluorescence. Cell proliferation and clonal formation experiments verified the functional effects of micropeptide on colon cancer cells. Co-immunoprecipitation (co-IP), mass spectrometry, and bioinformatic analysis were used to determine the possible mechanism of action of the micropeptide.

**Results:**

The lncRNA *RBM26-AS1* could encode two micropeptides with different relative molecular masses. The RBM26-AS1 peptide promotes colon cancer cell growth and colony formation. Mechanistically, the RBM26-AS1 peptide may play a role in enhancing nucleocytoplasmic transport and protein processing in the endoplasmic reticulum, which may be different from the function of lncRNA itself. The inhibition of the RBM26-AS1 peptide impairs the proliferation ability of colon cancer cells.

**Conclusions:**

A previously unknown micropeptide hidden in lncRNA *RBM26-AS1* may contribute to colon cancer progression by enhancing nucleoplasmic transport and protein processing in the endoplasmic reticulum.

## Introduction

Colorectal cancer (CRC) is the third most common cancer worldwide and the second leading cause of cancer death^[1]^, placing great pressure on both prevention and treatment. Moreover, growing concern has been raised globally due to the continued increase in early-onset CRC (age ≤50 years) with unknown cause^[2]^. Despite the availability of standard chemotherapy, targeted therapy, and the ongoing exploration of immune checkpoint inhibition therapy^[2]^, the overall 5-year survival rate of CRC patients, especially those with advanced conditions, remains unsatisfactory. Therefore, a better understanding of the mechanisms of colon tumorigenesis could provide the opportunity to develop specific medicines that target specific mechanisms.

LncRNAs exhibit distinct expression patterns in diseases and have been identified as biomarkers or therapeutic targets^[3,4]^. Antisense lncRNAs are a class of lncRNAs derived from antisense transcripts of coding genes^[5]^. Functionally, lncRNAs play a regulatory role in a variety of pathophysiological contexts including development, metabolic process, cardiovascular system, immune regulation, and cancer progression through distinct molecular mechanisms such as chromatin structure regulation, molecular condensates formation, transcriptional regulation, etc.^[6]^ The mechanistic pathways of lncRNAs exhibit greater diversity than those of small non-coding RNAs, many of which remain poorly understood^[6]^. Hence, more efforts are warranted to unravel the functional mechanisms of lncRNAs. Pervasive translation of noncanonical open reading frames (ORFs) outside of canonical coding sequences has been revealed via Ribosome profiling^[7,8]^. These ORFs are capable of encoding micropeptides, which are generally less than 150 amino acids in length and show low evolutionary conservation^[9]^. It was previously thought that short peptides were difficult to fold into stable structures to perform specific functions. However, it has been shown that the micropeptides encoded by these ORFs often have distinct cellular localization and strongly influence the cell phenotype through specific mechanisms^[7]^. For example, lncRNA-derived micropeptides exert tumor-promoting or tumor-suppressive effects by regulating selective RNA splicing, RNA degradation, cellular senescence, mitophagy, and metabolism, opening a new world of proteins^[8,10]^. These add new insights into the complex role of lncRNAs. However, most lncRNAs contain small ORFs, whose translational potential and functional importance have not been validated so far^[11]^.

**HIGHLIGHTS**

- LncRNA *RBM26-AS1* can encode two micropeptides with different molecular masses.
- Micropeptide encoded by *RBM26-AS1* promotes the proliferation and clonal formation of colon cancer cells.
- Micropeptide may contribute to colon cancer progression by enhancing nucleoplasmic transport and protein processing in the endoplasmic reticulum.
- The function of the micropeptide may be different from that of lncRNA *RBM26-AS1* itself.

In previous studies, we performed nucleolar protein mass spectrometry to identify many different peptides. By screening for peptides longer than 50 amino acids and further selecting genes that are highly expressed in colorectal adenocarcinoma (COAD), we finally identified RNA Binding Motif Protein 26 Antisense RNA 1 (*RBM26-AS1*) as a candidate. *RBM26-AS1* is a lncRNA derived from the antisense strand of the encoding gene RNA Binding Motif Protein 26 (RBM26). Previous study has suggested that *RBM26-AS1* may be involved in the development and recurrence of acute pancreatitis through a ceRNA regulatory network involving *RBM26-AS1-hsa-miR-200b-3p-FHIT*^[12]^. Another study has also shown that the *RBM26-AS1*/*hsa-miR-182-5p*/*S100A2* ceRNA network is involved in the regulation of immune-related gene expression and contributes to the pathogenesis of autism spectrum disorder^[13]^. However, to the best of our knowledge, the role of *RBM26-AS1* in colon cancer has not been characterized.

In this study, we predicted and validated the coding potential of lncRNA *RBM26-AS1*, which was previously considered to be a non-coding gene, and explored the role and possible mechanism of *RBM26-AS1*-encoded micropeptide on colon cancer progression. This study uncovers that lncRNA promotes tumor development in a novel way by encoding the micropeptide, providing new insights into the pathogenesis of colon cancer. This study complies with the TITAN guidelines^[14]^.

## Materials and methods

### Cell lines

All cell lines were obtained from ATCC and verified by short tandem repeat assays for their identification, and cultured at 37°C in a cell incubator containing 5% carbon dioxide. The HCT116 cell was cultured in RPMI 1640 medium (Gibco) supplemented with 10% fetal bovine serum (FBS) (Gibco) and 1% penicillin/streptomycin (Gibco). The human embryonic kidney HEK293T cell and SW480 cell were cultured in DMEM medium (Gibco) supplemented with 10% FBS (Gibco) and 1% penicillin/streptomycin (Gibco).

### Plasmid constructs and lentiviral infection

EGFP with the start codon mutation (EGFPmut) was ligated to human *RBM26-AS1* ORF (ORF-EGFPmut) and the ORF with the start codon mutation (ORFmut) (ORFmut-EGFPmut), and then they were cloned into the pLVX-IRES-Neo lentiviral expression vector. To generate the FLAG fusion protein, FLAG was ligated to the 3ʹ-terminus of human *RBM26-AS1* ORF (ORF-FLAG) and the ORF with the start codon mutation (ORFmut) (ORFmut-FLAG), and then they were cloned into the pLVX-IRES-Neo lentiviral expression vector. The corresponding sequences of primers are listed in the supplementary file. The lentiviral particles were generated in HEK293T cells by cotransfection of the target gene expression vector constructs indicated above, pMD2.G and psPAX2. The control virus particles were produced by cotransfection of pLVX-IRES-Neo expression vector without target genes, pMD2.G and psPAX2.

### RNA extraction and quantitative reverse transcription-PCR analysis

RNA was isolated using RNAiso Plus (TaKaRa). 1 ml of trizol was added directly to the cells in the culture dish. The Trizol solution containing cells was transferred into a 1.5 ml ribonuclease-free centrifuge tube. After 10 min at 12 000 rpm at 4° C, the supernatant was removed and transferred into a new EP tube. Chloroform was added for extraction. The aqueous phase RNA was precipitated by the addition of isopropanol. The RNA precipitate was washed by adding 75% ethanol. Finally, the RNA was quantified. Standard cDNA was generated using PrimeScript® Reverse Transcriptase Master Mix (TaKaRa) following the manufacturer’s protocol. Quantitative real-time PCR (qRT-PCR) was performed on QuantStudio 7 Flex Real-Time PCR System (Thermo Fisher) using SYBR Premix Ex Taq (TaKaRa). GAPDH was used as a control, and the relative gene expression was calculated using the comparative cycle threshold (Ct) (2^−∆∆Ct^) method.

### Western blotting

Cells were lysed in a lysis buffer containing 1% protease inhibitor to obtain the total cell lysates. Protein concentrations were measured using a BCA Protein Assay Kit (Beyotime Technology). Total proteins were separated by 15% sodium dodecyl sulfate polyacrylamide gel electrophoresis (SDS–PAGE) and transferred to polyvinylidene fluoride (PVDF) membranes (Millipore). The membranes were blocked with a blocking solution prepared with TBST containing 5% skim milk for 1 h at room temperature and then incubated with primary antibody overnight at 4°C. The membranes were washed and incubated with HRP-conjugated secondary antibody (Cell Signaling Technology) for 1 h at room temperature. Finally, visualization was performed using the ChemiScope 6000 Touch Imaging system (Clinx, SHH, China).

### Immunofluorescence

The appropriate number of cells was spread in a confocal dish. The cells were fixed with 4% paraformaldehyde for 15 min at room temperature and then permeabilized with 0.5%Triton X-100 (prepared in 1× PBS) for 20 min at room temperature. A blocking buffer containing 1% donkey serum was added and blocked for 1 h at room temperature. Subsequently, the blocking solution was removed by aspiration and a sufficient amount of diluted primary antibody was added and incubated overnight at 4°C. The next day, the primary antibody was removed, and the cells were washed three times with PBST (1× PBS containing 1‰Tween20), then the diluted fluorescent secondary antibody was added and incubated for 1 h at room temperature. The secondary antibody was removed and DAPI was added and incubated in the dark for 5 min to stain the nuclei. Finally, the images were observed under a fluorescence microscope and acquired.

### Anti-RBM26-AS1 antibody preparation

Micropeptide synthesis and anti-RBM26-AS1 antibody preparation were performed by GL Biochem (Shanghai) Ltd. Peptide sequences KTLFLTRRKRPLTSETAGTPARPR were synthesized and inoculated into rabbits to produce polyclonal antibodies against RBM26-AS1 peptide. Subsequently, antibodies were purified using affinity chromatography columns.

### Nuclear and cytoplasmic separation assay

Briefly, SW480 cells were collected and mixed with PBS, and a portion was removed into a new EP tube. After lysis with RIPA buffer on ice, some of the cell lysates were removed into new EP tubes for western blot analysis. The remaining supernatant was used for total RNA extraction. Cells in another EP tube were centrifuged after buffer A was added, and the supernatant was collected and labeled cytoplasmic. Buffer A was added to the precipitate and labeled as the nucleus, the supernatant was then discarded by centrifugation, and buffer S1 and buffer S2 were added to the precipitate in turn. The EP tubes labeled as Cytoplasmic were centrifuged and some were removed for RNA extraction, another portion was used for western blot analysis. Cells in EP tubes labeled nucleus were lysed by adding RIPA buffer. Part of the lysate was removed for western blot assay, and the remainder was used directly for RNA extraction.

### CellTiter-Glo® Luminescent cell viability assay and colony formation assays

Cells were plated in 96-well plates at a density of 8 × 10^2^ cells per well. Cell proliferation was quantified with the CellTiter-Glo® Luminescent Cell Viability Assay Kit and measured every other day with a multi-mode microplate reader (Syner gy HTX, BioTek, Winooski, United States). For the colony formation assays, Adherent cells were digested into single cells by trypsin and suspended in culture medium for later use. After cell counting, 800–1500 cells per well were added to a 6-well dish according to the speed of cell proliferation, and the cells were gently shaken to disperse evenly. The cells were cultured in a 5% CO_2_ cell incubator at 37℃ for 1–2 weeks. Cultures were terminated when macroscopic clones appeared. Two milliliters of fixative solution (methanol: acetic acid = 3:1, volume ratio) was added to fix the cells for 1 h. Then the fixative was removed and the cells were stained with an appropriate amount of crystal violet staining solution for 10 to 30 min. The staining solution was then removed, the plate was inverted, and clones were counted directly by eye.

### Co-immunoprecipitation and mass spectrometry

The appropriate amount of cells transfected with the ORF-FLAG fusion plasmid were collected and lysed with the appropriate amount of co-IP lysis buffer for 30 min on an ice shaker. The supernatant was collected and the concentration was measured, and part of the supernatant was removed as input. An appropriate amount of flag magnetic beads was added to the protein lysate and mixed overnight with a 4° rotation. The next day, an appropriate amount of IP buffer was added to wash the magnetic beads several times. The 3 × flag eluate was added and rotated at 4° for 2 h. Loading was added to the supernatant and protein was boiled. Subsequently, equal amounts of the protein product obtained with co-IP were sampled on 15% SDS-PAGE. Then the gel was stained using a rapid silver staining kit (Beyotime) following the manufacturer’s instructions. After silver staining, the experimental groups were sent for mass spectrometry.

### Data and visualization

*RBM26-AS1* expression in COAD and its different stages, as well as genes with similar expression patterns to *RBM26-AS1*, were derived from The Cancer Genome Atlas (TCGA)-based data^[15]^. The Genotype-Tissue Expression (GTEx) data were obtained from UCSC Xena (https://xena.ucsc.edu/). Gene Ontology (GO) annotation and Kyoto Encyclopedia of Genes and Genomes (KEGG) analysis were performed according to previously described methods^[16]^.

### Statistical analysis

Data were expressed as mean ± standard deviation (SD). Statistical analysis was performed using Prism 8 software (GraphPad Software, Inc.) and R software (version 4.4.1). Two-tailed Student’s *t*-tests as well as one-way ANOVA were performed to calculate the statistical significance of the differences between groups. The categorical data were analyzed using the chi-squared test. A *P*-value of less than 0.05 was considered statistically significant. ns, no significant, *P < 0.05, **P < 0.01.

## Results

### *Characterization of lncRNA* RBM26-AS1 *in colon cancer*

**Figure 1.**
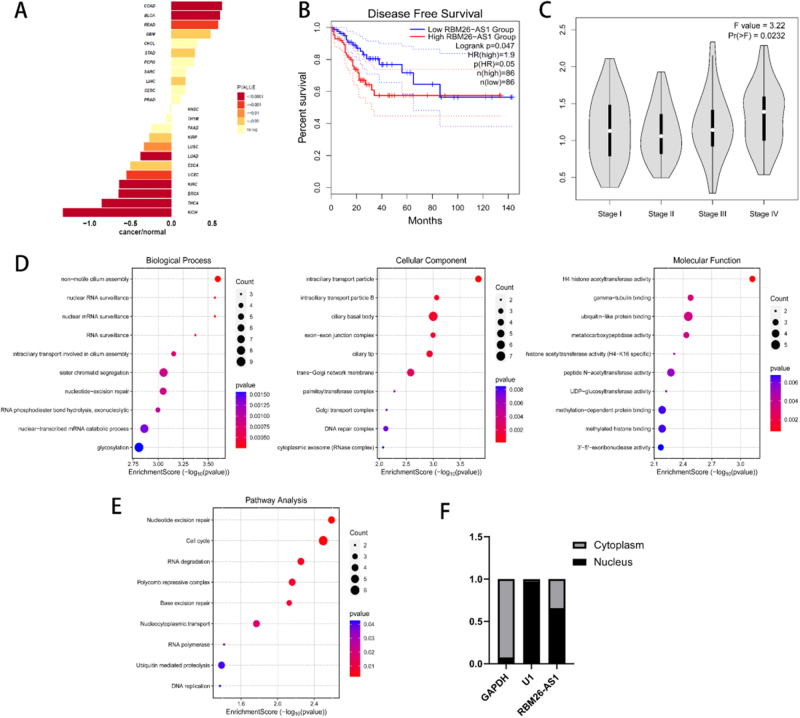
Characterization of lncRNA *RBM26-AS1* in colon cancer. (A) Expression level of *RBM26-AS1* in TCGA common cancers compared to normal tissues. (B) Comparison of disease-free survival between low *RBM26-AS1* group and high *RBM26-AS1* group (The sample size of both the low group and the high group was 86, *P* < 0.05). (C) Expression levels of *RBM26-AS1* in different stages of COAD (*P* < 0.05). (D) GO enrichment analysis of genes related to *RBM26-AS1*, including biological process, cellular component, and molecular function. (E) KEGG pathway analysis of genes related to *RBM26-AS1*. (F) Nucleoplasmic dissociation assay for *RBM26-AS1.*

According to TCGA data, the log2 fold change of *RBM26-AS1* expression in cancer tissues relative to normal tissues indicated that the expression of *RBM26-AS1* was the highest in colon adenocarcinoma (COAD) (Fig. 1A). The data from GTEx also indicated that the expression of *RBM26-AS1* was upregulated in colon cancer (Supplementary Digital Content Figure S1, Available at – http://links.lww.com/JS9/E634). The high *RBM26-AS1* group showed poorer disease-free survival compared to the low *RBM26-AS1* group (Fig. 1B), although it may not be related to overall survival (Supplementary Digital Content Figure S2, Available at – http://links.lww.com/JS9/E634). With the increase of the COAD stage, the expression level of *RBM26-AS1* also gradually increased (Fig. 1C). Clinical analysis indicated that the expression of *RBM26-AS1* might be higher in female patients compared to male patients (Supplementary Digital Content Table S1, Available at – http://links.lww.com/JS9/E634). To discover the possible functional role of lncRNA *RBM26-AS1* in tumors, the genes with similar expression patterns to *RBM26-AS1* in COAD were obtained through GEPIA2, where these genes were ranked by Pearson correlation coefficient^[15]^. GO annotation and KEGG functional analysis showed that *RBM26-AS1* might be involved in RNA surveillance, H4 histone acetyltransferase activity, and nucleotide excision repair (Figs. 1D and 1E). The nucleoplasmic separation assay demonstrated that *RBM26-AS1* might be mostly located in the nucleus (Fig. 1F). This might be consistent with its function. Together, these data indicate that *RBM26-AS1* may have a functional role in COAD.

### LncRNA RBM26-AS1 has the potential to encode micropeptides

**Figure 2.**
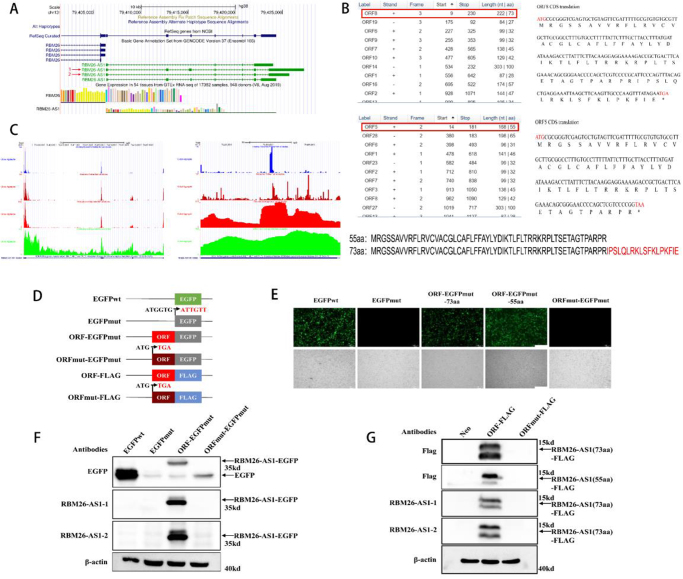
Prediction and validation of the coding potential of *RBM26-AS1*. (A) RBM26-AS1 has a total of four transcripts, and the red arrows indicate the transcripts referred to in the study. (B) The predicted ORFs and their encoded micropeptide sequences, as well as the sequence alignment of the two micropeptides. (C) Ribosome profiling of the predicted ORFs was performed based on the GWIPS-viz database. (D) Schematic representation of the EGFP fusion constructs and the FLAG fusion constructs. The start codon of EGFP was mutated from ATGGTG (EGFPwt) to ATTGTT (EGFPmut). The start codon of ORF was mutated from ATG to TGA (ORFmut). EGFPmut was fused to the c terminus of ORF and ORFmut to construct ORF-EGFPmut and ORFmut-EGFPmut fusion constructs. FLAG was fused to the C-terminus of ORF and ORFmut to construct ORF-FLAG and ORFmut-FLAG fusion constructs. (E–G) The indicated constructs were transfected into HEK293T cells and SW480 cells for 48 h. EGFP fluorescence was detected (E). The ORF-EGFP fusion protein (F) was detected by immunoblotting with anti-EGFP antibody and anti-RBM26-AS1 peptide antibody. The ORF-FLAG fusion protein (G) was detected by immunoblotting with anti- FLAG antibody and anti-RBM26-AS1 peptide antibody.

*RBM26-AS1* was annotated as a noncoding RNA. However, the ribosome profiling indicated that the *RBM26-AS1* transcript was bound by the ribosome, exhibited translational potential, and might encode a micropeptide. *RBM26-AS1* had four transcripts (Fig. 2A). By using the ORF finder tool, we further found that a 168-nucleotide small ORF in the first transcript was capable of encoding a 55-amino acid (55aa) micropeptide. Moreover, a 222-nucleotide small ORF in the second transcript could encode a 73-amino acid (73aa) micropeptide (Fig. 2B). The amino acid sequences of the 2 micropeptides were identical for the first 55 amino acids, except for additional 18 amino acids in the second micropeptide (Fig. 2B). Ribosome profiling of the ribosomal occupancy of the two ORFs based on the data from the GWIPS-viz database showed significant peaks at initiating ribosomes (P-site), elongating ribosomes (A-site), footprints, and mRNA-seq reads, indicating the coding potential of the ORFs (Fig. 2C). Sequence alignment of these two micropeptides did not reveal homology to any other proteins, denoting that they might be uncharacterized protein products.

### LncRNA RBM26-AS1 encodes micropeptides

To verify the coding ability of *RBM26-AS1*, several constructs were generated. Wild-type EGFP (EGFPwt) was mutated to mutant EGFP (EGFPmut) (the start codon was changed from ATGGTG to ATTGTT). The start codon ATG of both ORFs was mutated to TGA (ORFmut). Then, the EGFPmut was fused to the C-terminus of each of these two ORFs, and EGFPmut was also fused to the C-terminus of ORFmut, respectively (Fig. 2D). Abundant expression of the ORF-EGFPmut fusion protein was observed in transfected cells under fluorescence microscopy. However, the mutation of the ORF start codon (ORFmut-EGFPmut) abolished the expression of the ORF-EGFP fusion protein (Fig. 2E), demonstrating that the start codon of ORF could initiate translation. Western blotting assays using anti-EGFP antibodies further confirmed the expression of the ORF-EGFP fusion protein at positions of the predicted relative molecular mass, whereas ORFmut-EGFPmut abolished the expression of the ORF- EGFP fusion protein (Fig. 2F).

Since the EGFP tag is much larger than the small RBM26-AS1 micropeptide, to exclude the possible effect of the EGFP tag on the micropeptide, we generated fusion proteins with the FLAG tag to replace the EGFP tag. The FLAG tag was fused to the C terminus of the ORF and ORFmut (Fig. 2D). Subsequently, western blotting assays using anti-FLAG antibodies further confirmed the expression of the ORF-FLAG fusion protein at positions of the predicted relative molecular mass, whereas ORFmut-FLAG did not (Fig. 2G). These results were consistent with those of the ORF-EGFP fusion protein. Therefore, the ORF-FLAG fusion construct was used in the following experiments.

Furthermore, the antibodies against the RBM26-AS1 peptide were produced to detect the micropeptide encoded by the ORF in cells. Western blotting analysis with anti-RBM26-AS1 antibodies was performed to detect the ORF-FLAG fusion protein expression in cells transfected with different FLAG constructs. The results showed that the fusion protein could be detected in cells transfected with RBM26-AS1 ORF-FLAG but not in cells transfected with ORFmut-FLAG with a mutation in the start codon of the ORF (Fig. 2G). Consistent results were also detected for the RBM26-AS1 ORF-EGFP fusion protein (Fig. 2F). Together, these data confirm that the RBM26-AS1 fusion proteins were expressed in cells.

### The micropeptide encoded by RBM26-AS1 promotes colon cancer cell proliferation and colony formation

**Figure 3.**
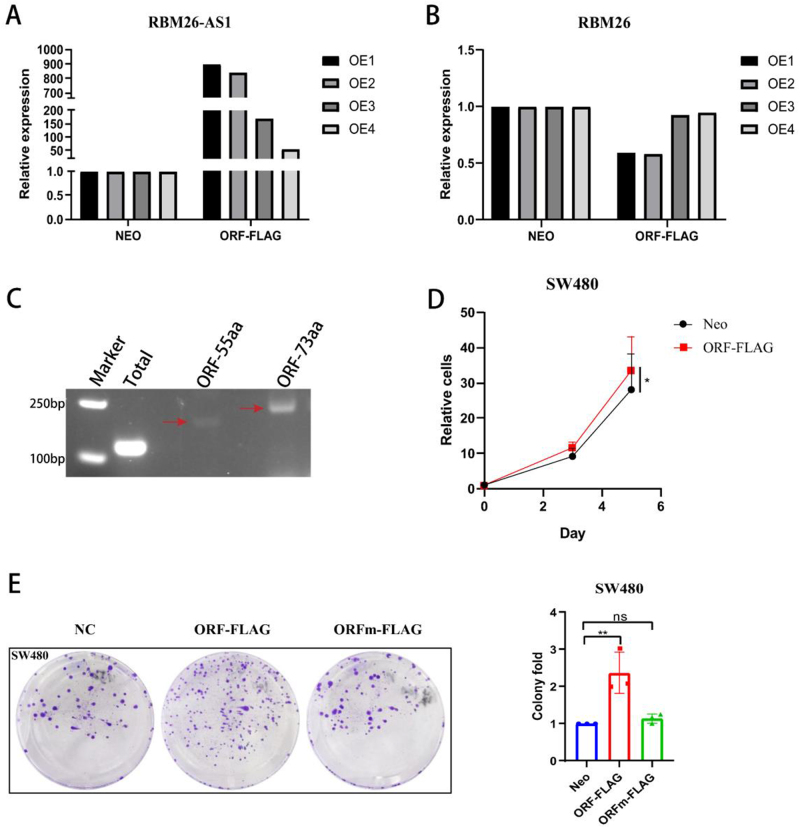
The micropeptide encoded by RBM26-AS1 promotes colon cancer cell proliferation and colony formation. (A) SW480 cells were transfected with the indicated constructs, and the overexpression fold of RBM26-AS1 ORF-FLAG was analyzed by qRT-PCR. (B) SW480 cells were transfected with the indicated constructs, and the corresponding effects of the RBM26-AS1 ORF-FLAG on the RBM26 gene at different overexpression multiples were analyzed by qRT-PCR. (C) Intracellular levels of the two ORFs were detected by RT-PCR. Red arrows indicate where two different ORFs are located, respectively. (D) SW480 cells were transfected with the indicated constructs, and the number of cells at the indicated time points was measured (*n* = 3) (*P < 0.05). (E) SW480 cells were transfected with the indicated constructs and their clonogenic ability was examined (*n* = 3) (**P < 0.01).

Given that *RBM26-AS1* was transcribed from the antisense strand, the level of the sense strand gene *RBM26* was further detected. The results showed that as the ORF overexpression fold decreased (Fig. 3A), the effect on the *RBM26* gene also gradually alleviated (Fig. 3B). As a result, to exclude the possible influence of *RBM26* gene changes, the cells with low overexpression fold were used for subsequent functional and other tests.

In addition, we detected the amount of intracellular expression of these two ORFs at the background level. The results indicated that the expression level of the ORF encoding 73aa was significantly higher than that of the ORF encoding 55aa, and the ORF encoding 73aa was the first and most easily amplified in the initial RT-PCR (Fig. 3C). The 73aa micropeptide was therefore used in subsequent experiments.

To determine the functional role of the RBM26-AS1 peptide on colon cancer progression, the *RBM26-AS1* ORF-FLAG and ORFmut-FLAG constructs were transfected into colon cancer cells. The *RBM26-AS1* ORF-FLAG construct capable of expressing RBM26-AS1 peptide promoted colon cancer cell proliferation and colony formation (Figs. 3D and 3E). The *RBM26-AS1* ORFmut-FLAG construct, which was unable to encode the RBM26-AS1 peptide because the start codon of the ORF had been mutated, did not affect colon cancer cell proliferation and colony formation (Figs. 3D and 3E). Taken together, these data indicate that the *RBM26-AS1* gene-encoded micropeptide, but not its lncRNA, plays a tumor promoter role by enhancing the proliferation and colony formation ability of colon cancer cells.

### The functional mechanisms of micropeptide

**Figure 4.**
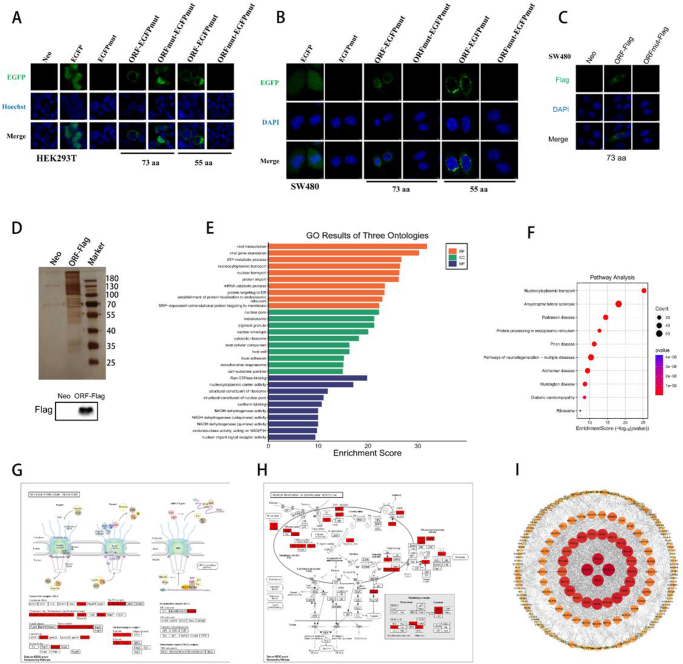
The functional mechanisms of micropeptide. (A) The indicated constructs were transfected into HEK293T cells, and EGFP fluorescence was detected in living cells. (B) The indicated constructs were transfected into SW480 cells, and EGFP fluorescence was detected in fixed cells. (C) The indicated constructs were transfected into SW480 cells, and ORF-FLAG fusion protein was immunostained with an anti-FLAG antibody. (D) The specific proteins that interacted with the RBM26-AS1 peptide. (E) GO analysis of proteins interacting with RBM26-AS1 peptide. (F) KEGG analysis of proteins interacting with RBM26-AS1 peptide. (G) Nucleocytoplasmic transport pathways in which proteins interacting with RBM26-AS1 peptide may be involved. (H) Proteins interacting with the RBM26-AS1 peptide may be involved in protein processing pathways in the endoplasmic reticulum. (I) Interactions between proteins interacting with the RBM26-AS1 peptide.

To further demonstrate the intracellular localization of the micropeptide encoded by *RBM26-AS1*, immunofluorescence assays were performed using anti-EGFP antibody, anti-FLAG antibody, and direct visualization of the ORF-EGFPmut fusion protein in living cells. The subcellular localization of the RBM26-AS1 ORF-EGFPmut fusion protein was observed by fluorescence confocal microscopy, however, the mutation of the ORF start codon (ORFmut-EGFPmut) abolished this subcellular localization (Figs. 4A and 4B). The apparent subcellular localization of the RBM26-AS1 ORF-FLAG fusion protein was also observed by immunofluorescence assay using an anti-FLAG antibody, however, this subcellular localization was abolished by mutation of the ORF start codon (ORFmut-FLAG) (Fig. 4C). The two modalities of observation demonstrated consistent subcellular localization. The micropeptide was located around the nuclear membrane and circled the nucleus. Collectively, these data suggest that the micropeptide has a specific subcellular localization.

To explore the possible functional mechanism of the micropeptide encoded by *RBM26-AS1*, the RBM26-AS1 ORF-FLAG construct was transfected into HEK293T cells, and then the RBM26-AS1-FLAG fusion protein was immunoprecipitated using anti-FLAG antibodies. The results showed many obvious specific bands compared with the control group (neo group) (Fig. 4D). Mass spectrometry (MS) was next performed. Subsequently, GO annotation, KEGG functional analysis, and protein–protein interaction (PPI) network were performed on the MS results to identify the functional roles of these proteins. GO analysis suggested that the micropeptide might be involved in the process of nucleocytoplasmic transport and protein targeting to the endoplasmic reticulum, constituting the components of the nuclear pore and nuclear envelope, and playing the role of Ran GTPase binding and nucleocytoplasmic carrier activity (Fig. 4E). KEGG analysis indicated that micropeptide might be involved in nucleocytoplasmic transport pathway and protein processing in the endoplasmic reticulum (Fig. 4F). The two relevant mechanistic pathways were listed along with the molecules involved (Figs. 4G and 4H). The PPI network demonstrated the possible interactions between these molecules, and SEC61A1 showed the highest degree of association with other proteins (Fig. 4I).

## Discussion

The role of RBM26-AS1 in colon cancer has not been explored, so we first obtain genes with similar expression patterns to RBM26-AS1 in COAD to anticipate its possible functional role. Functional analysis indicates that RBM26-AS1 may be involved in the regulation of RNA surveillance, H4 histone acetyltransferase activity, and nucleotide excision repair. This differs from the function of its hidden micropeptide as evidenced by our results. Micropeptides may play different functions from lncRNAs, which further proves the functional diversity of lncRNAs and many potential unknowns still need to be further explored.

Due to the presence of multiple transcripts, lncRNAs may encode multiple micropeptides of varying sizes according to different transcripts. In this study, the encoding potential of two ORFs in RBM26-AS1 was confirmed, which could encode two micropeptides with different sizes. However, despite this possibility, we confirmed that one was predominantly present and the other was probably present in small amounts, which also provided some reference for future studies. Although we have confirmed the possible role of the 73aa micropeptide, the 55aa micropeptide may have the same or different effects, and it might be worth our further investigation in the future. In addition, considering the possible transcriptional effect of antisense strand lncRNAs on sense strand genes^[17]^, we demonstrated that overexpression of lncRNA *RBM26-AS1* indeed exerted a suppressive effect on the *RBM26* gene. However, this inhibitory effect was abolished when the overexpression fold was low. This also guides us to control the appropriate expression fold in future studies.

Recently, more complex regulatory mechanisms of cancer malignant biology orchestrated by emerging non-coding RNA-encoded micropeptides have been uncovered to affect the progression of tumors, including CRC. For example, a conserved 53aa peptide encoded by the lncRNA *HOXB-AS3* inhibited splicing factor hnRNP A1-mediated pyruvate kinase M (PKM) splicing and subsequent metabolic reprogramming by competitively binding to the arginine residues in RNA-binding RGG box (RGG) motif of hnRNP A1, thereby suppressing colon cancer growth^[18]^. The lncRNA *LOC90024* encoded a 130aa small protein that facilitated the binding of splicing regulator serine- and arginine-rich splicing factor 3 (SRSF3) to the exon 3 of transcription factor Sp4 to induce the formation of the long Sp4 isoform and promote CRC tumorigenesis and progression^[19]^. Furthermore, a mitochondria-localized 94aa micropeptide encoded by *LINC00467* enhanced colorectal cancer cell proliferation by interacting with ATP synthase subunits α and γ to promote the construction and activity of ATP synthase^[20]^. These mechanistic insights place lncRNA-encoded micropeptides in a critical role in tumor progression and reconsider another novel functionally important way for lncRNAs to act.

Here, we report that a lncRNA *RBM26-AS1*-encoded micropeptide confers a selective proliferative advantage to colon cancer cells by enhancing protein processing in the endoplasmic reticulum and nucleocytoplasmic transport. The nuclear transport proteins-mediated nucleocytoplasmic transport of key macromolecular substances through the nuclear pore complex located on the nuclear envelope directly affects signal transduction, gene expression, development, and disease^[21]^. The importance of faithful nucleocytoplasmic transport is underscored by the role transport plays not only in tumor growth but also in drug resistance. Furthermore, aberrant nucleocytoplasmic transport of cell cycle regulators and tumor suppressor proteins may cause abnormal cell growth signals and apoptotic inactivation and lead to tumor development^[22]^. Importantly, nucleocytoplasmic transport is enhanced in colon cancer^[22]^. Among these molecules that may interact with micropeptide, SEC61A1/SEC61α (SEC61 translocon subunit alpha 1), a core component of the heterotrimeric SEC61 complex, plays a crucial role in the transport of secretory proteins and transmembrane proteins into the endoplasmic reticulum^[23,24]^. Moreover, the highly expressed SEC61A1 promoted the proliferation, migration, and invasion of colon cancer cells through the *MNX1-AS1*/*miR-218-5p*/*SEC61A1* axis^[25]^. Therefore, the precise mechanism of the RBM26-AS1 peptide in coordinating nucleocytoplasmic transport and protein processing in the endoplasmic reticulum may be worthy of more in-depth investigation in the future. In addition, *RBM26-AS1* is highly expressed in colon cancer and is associated with disease-free survival and disease stage. Therefore, *RBM26-AS1* may serve as a biomarker for COAD. RBM26-AS1 peptide promotes the progression of colon cancer. Therefore, inhibition of the RBM26-AS1 peptide has potential as a therapeutic strategy. Furthermore, it is expected that the combination of micropeptide inhibition with chemotherapy and immune checkpoint blockade therapy may produce a synergistic effect to inhibit the growth and metastasis of colon cancer. Meanwhile, future validation in normal colon epithelial cells will further support the cancer specificity of micropeptide.

## Conclusion

In summary, we found that lncRNA *RBM26-AS1*, which was annotated as a non-coding RNA, had the potential to encode two micropeptides with different relative molecular masses. The oncogenic role of *RBM26-AS1* is attributed to its micropeptide rather than the lncRNA itself. The RBM26-AS1 peptide may promote colon cancer cell growth by enhancing nucleocytoplasmic transport and protein processing in the endoplasmic reticulum. Inhibition of RBM26-AS1 peptide impairs the proliferation ability of colon cancer cells. This study provides new mechanistic insights into colon cancer progression and opens possible new therapeutic avenues. It is believed that in the future, more noncoding RNA-encoded micropeptides will be revealed regardless of disease type and continue to broaden the functional diversity of lncRNAs.

## Data Availability

The datasets used and/or analyzed in the current work can be obtained from the corresponding author upon reasonable request.
